# Imaging in COVID-19-related myocardial injury

**DOI:** 10.1007/s10554-020-02089-9

**Published:** 2020-11-19

**Authors:** Riccardo Cau, Pier Paolo Bassareo, Lorenzo Mannelli, Jasjit S. Suri, Luca Saba

**Affiliations:** 1 Department of Radiology, Azienda Ospedaliero Universitaria (A.O.U.), di Cagliari – Polo di Monserrato s.s. 554 Monserrato, 09045 Cagliari, Italy; 2 Mater Misericordiae University Hospital and Our Lady’s Children’s Hospital, University College of Dublin, Crumlin, Dublin, Republic of Ireland; 3 IRCSS SDN, Naples, Italy; 4 Stroke Monitoring and Diagnostic Division, ATHEROPOINT LLC, Roseville, CA USA

**Keywords:** COVID-19, Imaging, CMR, Myocarditis, Heart injury

## Abstract

Severe acute respiratory syndrome coronavirus 2 (SARS- CoV-2), previously named “2019 novel coronavirus” (2019-nCoV) is an emerging disease and a major public health issue. At the moment, little is known, except that its spread is on a steady upward trend. That is the reason why it was declared pandemic since March 11th, 2020. Respiratory symptoms dominate the clinical manifestations of the virus, but in a few patients also other organs are involved, such as their heart. This review article provides an overview of the existing literature regarding imaging of heart injury during COVID-19 acute infection and follow-up.

## Background

In December 2019, different pneumonia cases of unknown aetiology presenting with severe acute respiratory syndrome (SARS), occurred in Wuhan, Hubei Province, China [1, 2]. Since then, the disease has been spreading quickly from Wuhan to other geographical areas and countries. As to September 22th, 2020, 2,923,580 COVID cases in Europe were confirmed [3]. The SARS- CoV-2 has features which are typical of the coronavirus family. SARS-CoV-2 shares 82% genome sequence similarity to SARS-CoV and 50% genome sequence homology to Middle East respiratory syndrome coronavirus (MERS-CoV) [4]. Coronaviruses are quite common human pathogens, causing from mild acute respiratory disease (the common cold) to severe and potentially lethal respiratory tract infections [5]. A large number of reports provide descriptions of the clinical signs associated with COVID-19. Sun et al. observed that the most represented symptoms are fever and cough [6]. It is well known that different types of viruses, such as adenovirus, enterovirus and herpesvirus, but also coronavirus, may cause heart injury [7]; Alhogbani et al. reported that the MERS-CoV can cause acute myocarditis and heart failure [8]. Moreover, some patients with SARS may present with a transient increase in myocardial enzymes [9]. In previous report from China the prevalence of myocardial injury in COVID-19 was estimated to range between 23 and 28% [10, 11].

The incidence of cardiovascular complication remains unknown. Long et al. reported cardiovascular complications in the setting of COVID-19 pandemic [12]. In their report observed that myocardial injuries may occur in 7–31% patients, and around 7% of COVID related deaths were caused by myocarditis. Other cardiac complications observed were heart failure (23%) and arrhythmias (7%), while the incidence of acute coronary syndrome in COVID-19 patient is still unclear [12, 13]. This might be related to a marked reduction in percutaneous coronary artery interventions during COVID-19 pandemic, caused by lockdown effect [13]. Moreover, similarly to other viral illness, COVID-19 can trigger acute myocardial infarction and This might be due plaque rupture, coronary spasm or hypercoagulability with development of microthrombi [12, 13].

A challenge in coronavirus disease is represented by the occurrence of comorbidities, which may complicate patients’ outcomes. According to Wang et al. hypertension, diabetes, and cardiovascular diseases were the most common coexisting conditions. Furthermore, patients with underlying comorbidities were admitted to ICU and showed more signs of cerebrovascular accidents in comparison with patients who were non-comorbid [2].

The purpose of this review is to provide an overview of the existing literature regarding the imaging of heart injury during COVID-19 acute infection and follow-up.

## SARS-COV-2 and the cardiovascular system

Several studies hypothesize a potential role of cardiac damage induced by this virus. This is particularly important because of the link between lung and heart function. Previous reports about COVID-19 showed a significant increase in cardiac lesion biomarkers, including Cardiac troponin I (cTnI), Creatine Kinase (CK), α-hydroxybutyrate dehydrogenase (HBDB), Lactate Dehydrogenase (LDH), and NT-proBNP [14–17] (Table 1). Taken together, these laboratory abnormalities suggest that SARS- CoV-2 infection may be related to a variable degree of myocardial injury. Not only, but the alterations are similar to those previously observed in patients with MERS-CoV [2].

**Table 1:** Previous reports about myocardial necrosis marker in COVID-19 patients

	Patients with SARS-CoV-2 infection	Patients with abnormal cardiac biomarkers	Cardiac lesion biomarkers	Notes
Xu et al. [16]	53	30	LDH CK Myo TNT-HSST NT-proBNP	This study shows that cardiac abnormalities including elevated myocardial enzyme levels (56.6%) are common in COVID-19 patients
Wu et al. [5]	188	Abnormal hs-TNI 11.2% 68.6% LDH abnormal 76.1% α-HBDH abnormal Abnormal CK 11.2% Abnormal CK-MB 10.1%	hs-TNI CK CK-MB LDH α-HBDH	This study assessed the associations between heart injury indicators and mortality in COVID-19 patients and that high hs-TnI on admission can be associated with higher mortality
Bo Zhou et al. [26]	34	Abnormal c-TNI 8/8 in very severe group and 1/26 in severy group	c-TNI CK LDH α-HBDH	They found high percentage of increased cTnI levels in very severe COVID-19
Huang et al. [15]	41	Abnormal CK 13/40 (33%) Abnormal hs-TNI 5/41 (12%) Abnormal LDH 29/40 (73%)	LDH CK Hs-TNI	They report a cohort of 41 patients with laboratory confirmed 2019-nCoV infection
Chen et al. [29]	120	Abnormal c-TNI (n = 12, 10%) Abnormal NT-proBNP (n = 33, 27.5%)	NT-proBNP c-TNI	This study has shown condition of some patients with severe SARS-CoV-2 infection, patients might deteriorate rapidly a possible exitus was a fulminant myocarditis

A case series of 138 hospitalized patients with a COVID-pneumonia, all with pulmonary infiltrates on chest CT, showed that the most common complications among these patients included ARDS and arrhythmia in 20% and 17% respectively, as well as shock (9%) and acute cardiac injury (7%). Patients admitted to ICU were more likely to suffer from one of these complications than those treated in ward [2].

Shi et al. assessed the association between cardiac injuries and mortality in patients with COVID-19. They observed that patients with myocardial injuries were older, had more comorbidities, complications were more common, and mortality higher compared to those without cardiac damage. In addition, they reported that COVID-19 patients with preexisting cardiovascular diseases were more vulnerable to cardiac damage [14].

Nevertheless, there is still a lack of large multicentre studies and little evidence to establish a direct correlation between myocardial injuries and cardiovascular comorbidities.

COVID-19 could lead to cardiac injury indirectly (i.e. secondary to respiratory failure or to a harmful immune system response) or directly owing to viral replication in the myocardium, although the specific mechanism is still uncertain [14, 18].

In this respect, recently it was suggested that COVID-19 may enter myocardial cells simply binding type 2 ACE receptors on their surface [19].

Varga et al. showed the presence of viral elements within endothelial cells, which also expresses ACE 2 receptors on their surface. This finding may explains the possible development of endothelitides, such as Kawasaki or Kawasaki-like disease in children, as consequence of the virus entering the endothelial cells [20, 21]. In addition, it might explain also the susceptibility to acute myocardial injury especially in patients with pre-existing endothelial disorders. Unfortunately, the available scientific evidence about histology in COVID-19 patients is poor. Also the drugs, which are administered as a therapy against COVID-19, may have potentially harmful cardiovascular side-effects and interactions with other medications [22, 23].

Current literature reports hypothesize that SARS-CoV2 infection could lead to cardiovascular complications or exacerbate a pre-existing cardiovascular disease [24–28]. At the beginning of 2020, a Chinese case report described for the first time a patient with COVID-19 and cardiac comorbidities who passed away because of a fulminant myocarditis (FM) [18].

In addition, another study by Chen et al. reported that some deaths in SARS-CoV-2-infected patients were associated with the sudden appearance of a myocarditis. In patients with an evident cardiac injury, plasma IL-6 levels were dramatically increased, since notoriously cytokine storm plays a pivotal role in FM pathophysiogy [29].

## Role of imaging

Definitive COVID-19 diagnosis requires a positive reverse transcription-polymerase chain reaction test [30]. According to the current best medical practice suggestions, the diagnosis cannot be made by chest computed tomography; the latter, however, may be useful in assessing for a possible COVID-19 pneumonia, which usually is bilateral and with basal or multi-lobar distribution. Quickly progressive ground glass opacities, sometimes with consolidation, are the typical features. However, chest imaging has limited sensitivity for COVID-19, since up to 18% of the patients show normal chest-X ray or CT when their symptoms are mild, but this decreases to 3% in severe cases [31, 32].

**Fig. 1:**
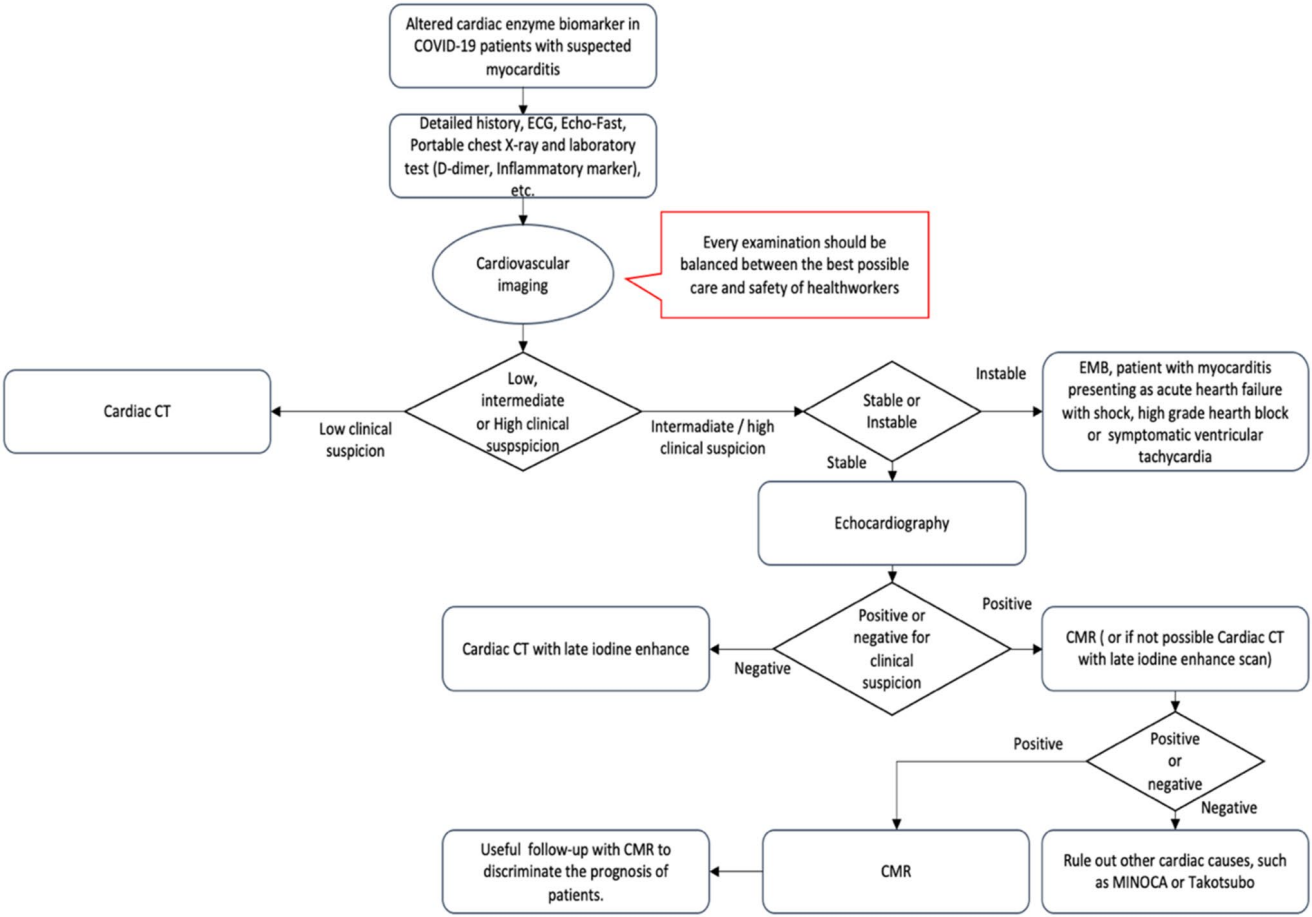
Diagnostic pathway for suspected myocarditis

As to cardiac involvement, non-invasive cardiac imaging during the pandemic might have an important role to decipher the rise in cardiac enzymes. In fact, the diagnosis of myocarditis remains still a challenge due to its variable clinical manifestations [33, 34] (Fig. 1).

The diagnostic gold standard for the diagnosis of myocarditis is endomyocardial biopsy (EMB) [35]. There are still no specific reports in literature about EMB in patients COVID-19 positive, except for a recent Chinese case report where a few interstitial mononuclear inflammatory infiltrates where described at a COVID-19 patient’s heart specimen examination [36]. The histological findings in COVID-19 greatly resemble those previously seen in SARS and Middle Eastern respiratory syndrome (MERS) coronavirus infection [37, 38].

In a position statement from the European Society of Cardiology (ESC) Working Group on Myocardial and Pericardial Diseases, EMB is strongly recommended. It should be done early in the course of the disease and multiple specimens should be taken in order to optimize its diagnostic accuracy and reduce sampling error in focal myocarditis [39, 40]. However, this procedure is infrequently used in clinical practice [35, 39–41].

The usual limitations of EMB in diagnosing myocarditis are represented by sampling errors caused by focal or patchy involvement of myocardium as well as high inter-observer variability in interpreting histopathological tissue [42]. Due to this uncertainty, several non-invasive imaging modalities are helpful in diagnosing myocarditis (Table 2) [39, 43–46].

**Table 2:** Non-invasive imaging in the diagnosis of myocarditis

Imaging modalities	Strengths	Limitations	Reccomandation during COVID-19 pandemic
Echocardiography	Safe Versatile Widely available technique No radiation exposure or use of contrast	Inadequate soft tissue characterization Poor acoustic windows Inter-observer variability Highly variable echocardiographic findings in myocarditis	Bedside echocardiography should be the first modalities in symptomatic COVID-19 patients with altered cardiac enzyme biomarker
CCT	High spatial resolution Tissue characterization	Radiation exposure Contrast medium reactions	Useful of CCT with late iodine enhance scan
CMR	Tissue characterization High spatial and temporal resolution Excellent reproducibility No radiation exposure	Low availability Costs Intrinsic or extrinsic factors of the patient (claustrophobia,metallic implants, allergy, ability to hold breath and arrhythmia) Long scan times	Main role in management of suspected myocarditis to confirm the diagnosis with fast CMR protocol
Nuclear medicine techniques	Marker of myocardial inflammation and necrosis	Limited specificity Cost Limited availability Radiation exposure	Not useful
Chest X ray	Indirect sign	Low sensitivity Very low specificity	Portable x-ray should be the first line modalities in asymptomatic and minimally symptomatic COVID-19 patients to have insight at the same time about lung and heart

In the same ESC position paper, it is recommended that all patients with clinically suspected myocarditis should undergo an echocardiogram at disease presentation. It should be repeated during hospitalization if there is any worsening in haemodynamic [39]. Indeed, echocardiography is a safe, versatile, and widely available technique. It allows us to evaluate and quantify global and regional systolic function and monitor any possible changes in cardiac chambers size, wall thickness, ventricular function, and pericardial effusion [39, 47, 48]. Echocardiography is the first-line imaging modalities, but at the same its diagnostic value is limited owing to the lack of specificity of a lot of echocardiographic findings. In addition, patients with myocarditis may have a normal echocardiogram as well [48, 49].

Echocardiography should not be performed routinely in patients with COVID-19, but bedside echocardiography could be a useful tool in clinical suspicion of myocardial involvement [45]. Moreover, the ventricular function can be evaluated in depth by using post- processing advanced echocardiography, such as myocardial strain. In patients with COVID-19 infection, Krishnamoorthy et al. reported that LV global longitudinal strain, RV global strain and free wall strain were altered. In addition, they observed that right ventricular strain may be important for risk stratification and prognosis as well. RV systolic dysfunction was more common than LV systolic dysfunction in COVID-19 patients. The etiology of this dysfunction could be related to pulmonary embolism, a common complication in this type of patients or to ARDS and its well-known sequelae, such as secondary pulmonary fibrosis [50, 51].

However, at present, there is still a lack of prospective study to assert with confidence the long- term pulmonary and cardiac consequences of COVID-19 [50, 51]. Echocardiographic myocardial strain is a time-consuming technique, a significant expertise is needed, and it is likely to be useful just in determining prognosis [45]. Other echocardiographic limitations include inadequate soft tissue characterization and suboptimal field-of-view in the setting of poor acoustic windows, such as in overweight/obese patients or in those suffering from SARS [52].

Conversely, nuclear imaging is not routinely recommended in the diagnosis of myocarditis [39, 42]. Sun et al. compared 99mTc-MIBI SPECT with other cardiac imaging techniques and assessed the presence of myocardial uptake of 99mTc-MIBI as a marker of myocardial inflammation and necrosis [53]. Limitations of this technique include its reduced specificity, high cost, limited availability, and radiations exposure [39]. Nuclear cardiology imaging requires long imaging acquisition time and specific protocols. Therefore, other safer and faster cardiovascular tests should be more useful during the current pandemic [54].

According to Dambrin et al. in suspected acute myocarditis the findings from ECG-gated multidetector CT correlated significantly with those observed at MRI examination [55]. Other studies confirmed the potential role of cardiac computed tomography (CCT) in the setting of myocarditis [56–58]. Pontone et al. suggested the use of CCT -with pre-contrast, contrast-enhanced, and delayed post-contrast scans- as a tool to detect myocardial scar and diagnose myocarditis when c-MRI is not feasible [59].

Given the rise of cardiac enzyme biomarker, CCT may play a leading role in ruling out other causes of cardiac damage, such as acute coronary syndrome [60].

It also allows an evaluation of extracardiac structures that could explain an increase in troponin values (for example, pericarditis). In fact, it can be used to study lung and aortic structures and exclude lung complication in COVID-19 patient such as pulmonary embolism, or cardiac complication such as ventricular dilation and intracavitary thrombi [10, 61].

The main limitation of CCT is radiation exposure for the patient. Therefore, CCT is considered useful when performing an MRI scan is not possible because of contraindications to MRI or suboptimal images due to artifacts [52].

Another non-invasive modality which, however, may play just a marginal role is chest X-Ray. It does not allow the diagnosis of myocarditis, but may show indirect signs, as cardiomegaly and/or pericardial effusion with low sensitivity (71%) and even less specificity (41%) [62, 63]. The American College of Radiology suggested the use of portable chest radiography as optimal tool to mitigate the risk of infection [64]. Baldirani et al. proposed a possible role for chest X-ray just for asymptomatic or minimally symptomatic patient in epidemic regions [65]. Therefore, in the setting of COVID-19 pandemic, (especially portable) chest X-ray might be the first line test in asymptomatic or with few symptoms patients to get information about lungs and heart dysfunction at the same time.

Cardiac magnetic resonance imaging (CMR), which provides non-invasive myocardial tissue characterization, is the gold standard imaging technique in diagnosing myocarditis [47]. Based on the Lake Louis criteria, CMR can identify myocardial damage with a diagnostic accuracy of 78%. Lake Louise criteria include detection of regional edema on T2-weighted CMR images, detection of hyperemia and early capillary leakage on the basis of T1-weighted early gadolinium enhancement, and detection of necrosis and fibrosis by late gadolinium enhancement (LGE), with high specificity and positive predictive value when 2 out of 3 CMR characteristics are present [48].

**Fig. 2:**
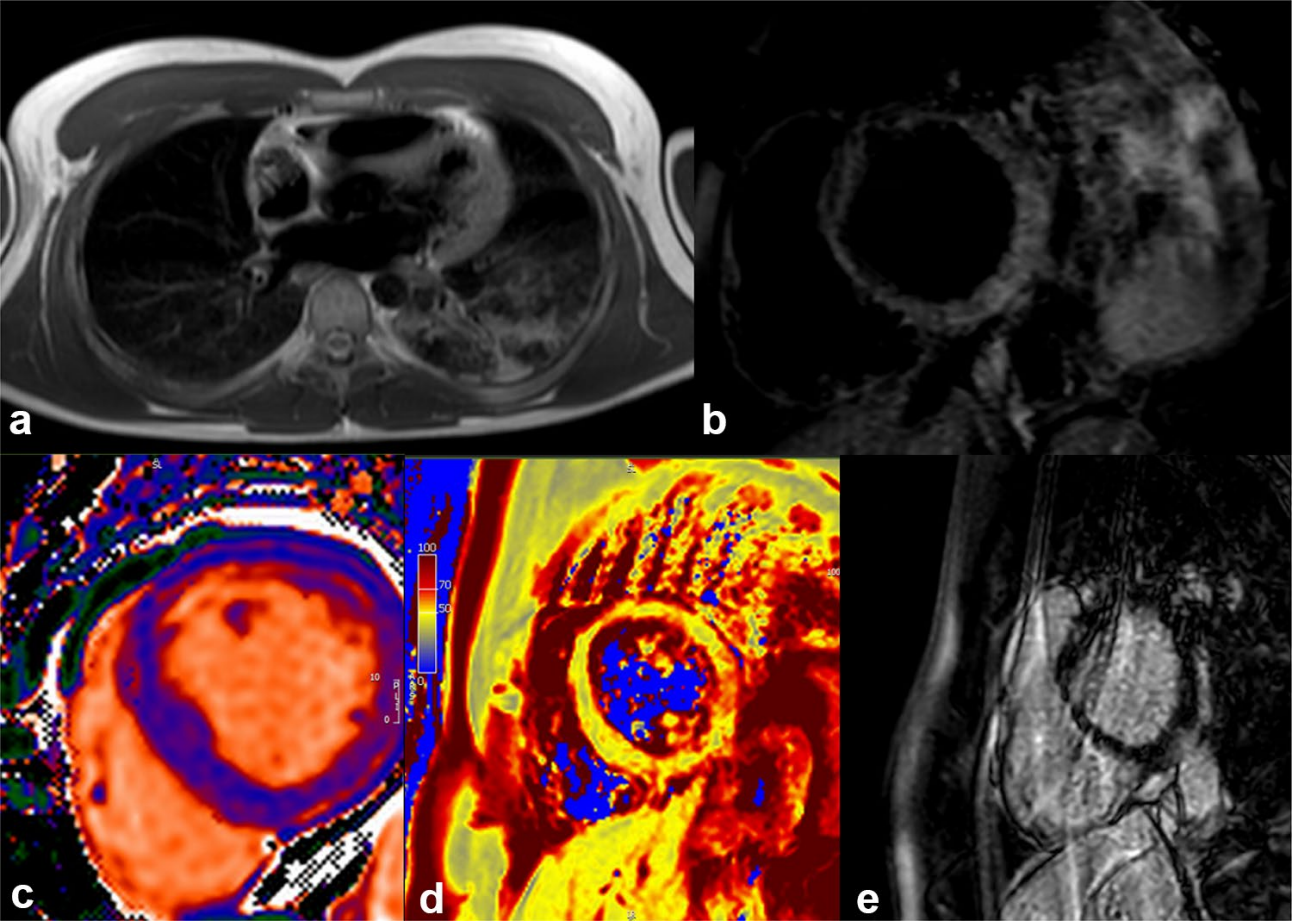
A 27-year-old patient, without any significant past medical history, was admitted to our hospital with fever and chest pain. The onset of symptomatology dated back about 1 week. His initial investigation showed elevated troponin levels at laboratory tests. Electrocardiography displayed ST-segment elevation. Viral myocarditis of unknown aetiology was initially suspected, but SARS-Co-V-2 as a cause was ruled out later at serology. Echocardiography was normal. A chest X-ray showed pulmonary consolidation at the left lower lobe. Cardiac magnetic resonance imaging confirmed the myocarditis (panel **a**). T2 STIR (panel **b**) showed an increased signal in mid-basal inferior and inferior-lateral segments. The analysis of T1 mapping (panel **c**) showed an increase in signal at the same segments (average values of 1100 ms, with reference values of 1030 ± 30 ms). T2 mapping values (panel **d**) showed an increased signal in mid-basal inferolateral segment (65 ms. Reference values: 52 ± 3 ms), thus indicating the presence of edema. In the sequences acquired later after contrast, an area of sub-epicardial LGE in mid-basal inferior and infero-lateral segments was observed with a concomitant involvement of the adjacent pericardium (panel **e**). Images processed with Circle CVI 42

Myocarditis-induced alterations may present with several patterns of LGE (Fig. 2), typically localized at the sub-epicardial and/or intramural regions of the left ventricle and frequently located in the basal to mid-inferolateral walls. Preliminary observation in the literature suggest that myocardial injuries might be related to other aetiologies than myocarditis. Guagliumi et al. suggested that patients with myocardial injuries and normal coronary artery might be caused by microvascular thrombosis in the absence of epicardial coronary obstruction [66]. Direct viral infection of the endothelial cell or vascular injuries caused by the virus might be explain impaired microcirculatory system with a prothrombotic state [12, 13, 20]. A literature review by Maiese et al. described autopsy findings in COVID-19 death. They have highlighted that SARS-CoV-2 causes endothelial dysfunction in various district [67]. Acute myocardial injuries might be due thrombotic complication [66, 68]. Therefore, the term myocarditis should be used with caution in COVID-19 patients with elevated troponin levels without a specific diagnostic test.

In this scenario, CMR features are crucial in making a differential diagnosis with myocardial infarction (sub-endocardial or trans-mural LGE distribution; regional wall motion abnormalities at SSFP sequencies vs global hypokinesia in myocarditis), takotsubo syndrome (diffuse myocardial wall oedema, without arterial territory distribution; transient mid-apical dyskinesis/akinesis; mild late gadolinium enhancement just in the areas of abnormal wall motion, and no LGE at follow-up after resolution), and MINOCA (i.e. myocardial infarction without non-obstructive coronary arteries, which is characterized myocardial oedema in a coronary distribution pattern [69].

**Fig. 3:**
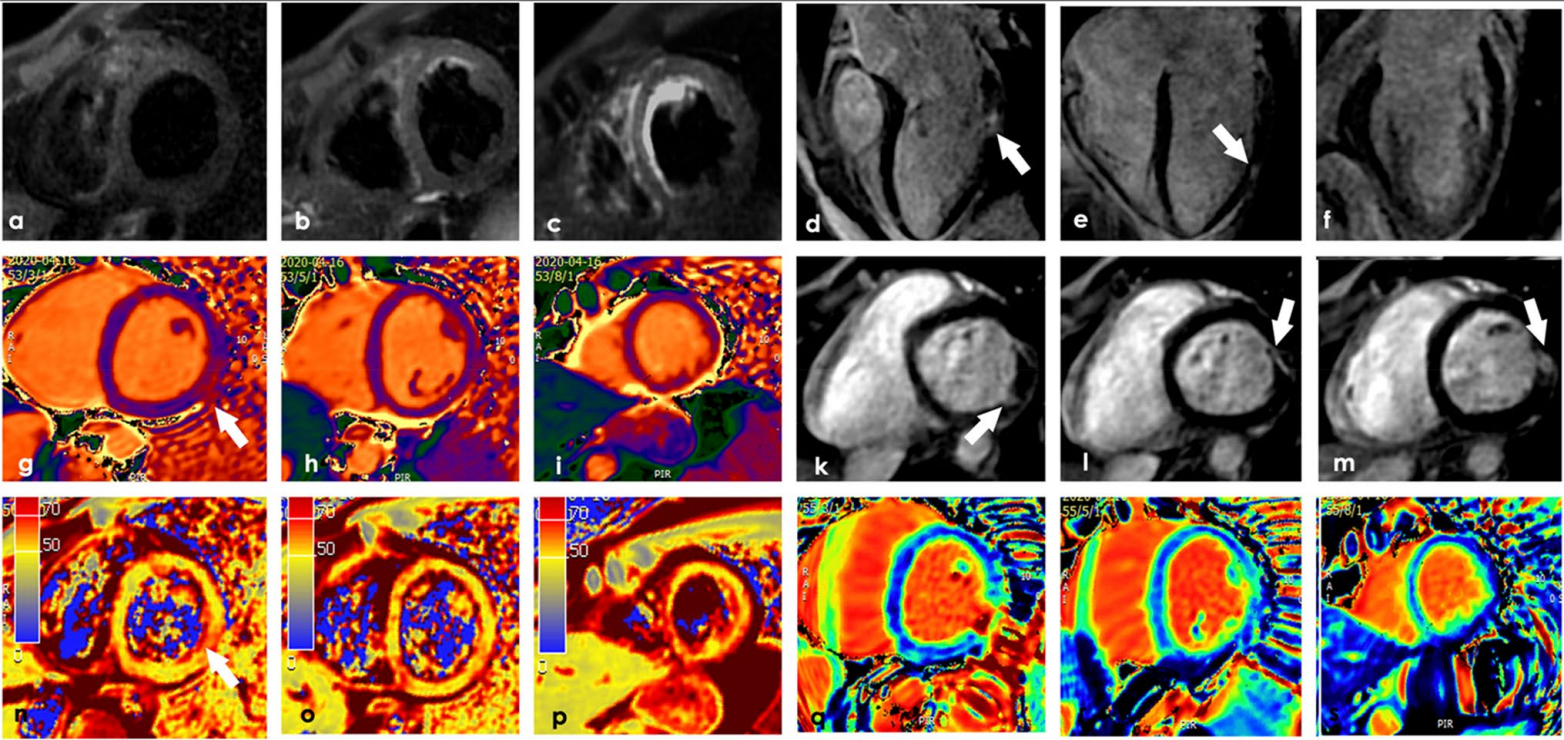
A 37-year-old male patient, without any significant past medical history, was admitted to our hospital with oppressive chest pain, sweating, severe fatigue. At the swab was positive for COVID19. Sublingual nitroglycerin showed no clinical benefits with alterations in the LV Sax TIR T2 sequences (**a**–**c**) with patchy alterations in the LAx MDE sequences (**d**–**f** white arrows). Also the native T1 mapping showed an area of alteration (white arrow) with areas of hypersignal in the SAx MDE sequences (**k**–**m**). The T2 map shows in the same region of the T1 map area of alteration (**n**–**p** white arrow). The **q**–**s** panels show the ECV. Courtesy of Professor Gianluca Pontone MD—Centro Cardiologico Monzino (Milano)

Additionally, LGE has proven prognostic value, because patients with areas of necrosis and fibrosis are at increased risk of adverse events [70], whereas LGE-negative patients have an excellent prognosis independently of their clinical symptoms [71]. CMR diagnostic accuracy could increase with the proposed updated Lake Louis criteria, which include parametric mapping techniques, such as T2 mapping, T1 mapping and ECV. Precisely, T2 mapping can identify acute myocardial edema and has several advantages compared with traditional T2-weighted imaging, among which higher signal-to-noise ratio and shorter breath-holds with fewer breathing motion artifacts. Again, native T1 is sensitive to intracellular and extracellular changes in free water content and its relaxation time increase during acute inflammation, vasodilation, and hyperemia (Fig. 3). Lastly, ECV detects an expanded extracellular space and compared with LGE may assess diffuse fibrosis and inflammation [37, 72].

Adding these parametric mapping techniques to the classic CMR protocol may improve its accuracy, provide additional disease characterization, and help management in different cardiac injuries [72, 73]. A recent research by Puntmann et al. including 100 patients recovered from COVID-19 infection, showed that native T1 and T2 mapping provide with the best differential parameters to detect COVID-19-related myocardial injuries [74] (Fig. 3).

CMR may be considered in clinically stable patients prior to EM [39]. Nonetheless, the latter should not be delayed in case of life-threatening presentations, because one of CMR limitations is that it is time-consuming, a long time being required for acquiring images (approximately 45 min with modern scanners) [39] (Table 3). A possible solution to reduce the examination time could be applying an ad hoc short protocol. So, Beitzke et al. proposed a fast CMR protocol, which incorporated reduced CINE sequences, parametric mapping, and LGE [11, 45]. Furthermore, CMR may be an additional diagnostic tool in myocarditis clinical follow-up, when detecting persistent or worsening LGE [75].

**Table 3:** CMR vs EMB

	EMB	CMR
Life-threatening presentations	+	−
Clinically stable patients	−	+
Arrhythmia	+	(−)
Onset of the disease is longer than 3 months ago	+	−
Follow-up	Non-responsive patients	+

LGE already proved to be one of the most important predictors of cardiovascular adverse outcomes in several cardiac conditions, myocarditis included [70, 76]. Thereby, in COVID-19 patients with suspected myocardial damage, CMR is the only non-invasive imaging modality that allows tissue characterization, myocardial inflammation detection, and reversible/irreversible injury assessment, thus evaluating the myocarditis activity and severity.

Currently, there is no evidence of possible long-term cardiac complications in COVID-19 patient, even though—as reported in recent research—an increased prevalence of arrhythmias and heart failure was detected [77–79].

CMR might play a leading role not only during the peak phase of the COVID-19 pandemic to decipher the cause of cardiac damage, but mostly afterwards, since it can reveal the presence of myocardial scar and consequent right and left ventricular remodeling that might influence the patient outcome [33, 51, 79].

Other possible, though less common, COVID-19 related cardiovascular manifestations are related to SARS-CoV-2 pro-thrombotic effects, with consequent risk of pulmonary embolism, myocardial infarction, and limb ischaemia [80]. Even in this setting cardiac imaging is proving its reliability (see Table 4). Collectively, SARS-CoV-2, cytokine release syndrome triggered by the viral antigen, drug-induced pulmonary toxicity, and high airway pressure secondary to mechanical ventilation cause secondary lung fibrosis in a considerable percentage of patients [81]. In the long-term, lung fibrosis is likely to cause the development of pulmonary hypertension and in turn *cor pulmonale*, i.e. a right sided heart failure. Even in this eventuality, cardiac imaging with echocardiography and CMR play a crucial role in providing valuable information about right ventricular mass, volume, kinesis, and function. By incorporating LGE, myocardial scar and fibrosis can also be evaluated by CMR.

**Table 4:** Role of imaging in other less common COVID-19-related cardiovascular manifestations

Pulmonary embolism	CT angiogram with contrast (identification of intra pulmonary artery/branches/pulmonary vessels clots) Echocardiography (right ventricle secondary involvement)
Myocardial infarction	Echocardiography (ventricular wall motion abnormalities) Coronary angiogram (stenosis identification, blood flow evaluation) IVUS (intra-vascular ultrasounds for plaque visualization)

## Conclusions

It is well known that viral infections may involve the heart as well (Table 5), inducing myocardial inflammation [40, 77] or other cardiovascular complications [12, 13, 20, 66], testified by an increase in cardiac enzymes, as well as a structural and functional damage. A few studies confirm that also SARS-CoV-2 can cause myocarditis and even congestive heart failure [21, 29, 65]. The higher the levels of cardiac enzymes and troponin, the higher the coronavirus mortality.

**Table 5:** Viruses that can cause myocarditis

RNA virus	Coxsackieviruses A and B, echoviruses, polioviruses, influenza A and B viruses, respiratory syncytial virus, Coronavirus, hepatitis C virus, dengue virus, yellow fever virus, human immunodeficiency virus-1
DNA virus	DNA viruses: adenoviruses, parvovirus B19, cytomegalovirus, human herpes virus-6, Epstein-Barr virus, varicella-zoster virus, herpes simplex virus, variola virus, vaccinia virus

Although the specific mechanisms are still a matter of concerns, an abnormal immune system response is likely to be the underlying cause of myocardial injury during coronavirus infection. The pandemic spread of the virus, as testified by the number of infected subjects getting higher and higher as times goes by, suggests that potential cardiac involvement should be identified early in view of a prompt diagnosis capable of improving patients’ outcome. In this respect, imaging—and in particular cardiac MRI with its most recent advanced tools—plays a crucial role. According to the International Guidelines, the involvement of a Heart Team made up of Radiologists and Cardiologists is critical in releasing a timely diagnosis, whose accuracy may help to save many lives threatened by such an aggressive disease.

## Abbreviations

SARS- CoV-2: Severe acute respiratory syndrome coronavirus 2
2019-nCoV: 2019 Novel coronavirus
SARS: Severe acute respiratory syndrome
MERS-CoV: Middle East respiratory syndrome
CK: Creatine kinase
CKMB: Creatine kinase-MB
cTnI: Cardiac troponin I
HBDB: α-Hydroxybutyrate dehydrogenase
LDH: Lactate dehydrogenase
EBM: Endomyocardial biopsy
ESC: European Society of Cardiology
CMR: Cardiac magnetic resonance
NT-proBNP: N-terminal pro B-type natriuretic peptide
IL-6: Interleukin 6
FM: Fulminant myocarditis
99mTc-MIBI SPECT: Technetium-99m-labelled methoxyisobutyl isonitrile SPECT
CCT: Cardiac computed tomography
LGE: Late gadolinium enhancement
